# Three-channel ion chromatograph for improved metabolic evaluation of urolithiasis

**DOI:** 10.1186/s12894-021-00914-4

**Published:** 2021-11-06

**Authors:** Qiang Li, Guanlin Liu, Yue Cheng, Wenbo Tang

**Affiliations:** 1 Department of Urology, Ningbo First Hospital, Zhejiang University, Ningbo, Zhejiang People’s Republic of China; 2 Department of Gynecology, Ningbo Women and Children’s Hospital, Zhejiang University, Ningbo, Zhejiang People’s Republic of China

**Keywords:** Citrate, Ion chromatograph, Metabolic evaluation, Oxalate, Urinary calculus, Urolithiasis

## Abstract

**Background:**

Urolithiasis is a multi-etiological disease resulting from a combination of environmental and genetic factors. One of the most challenging aspects of this disease is its high recurrence rate. For most patients, an in-depth metabolic evaluation may reveal the presence of urinary stones. The fact that different urinary stone-related compounds (USRCs) are measured by different methods renders the metabolic evaluation of urolithiasis quite tedious and complex.

**Methods:**

A three-channel ion chromatograph (IC) that automatically measures the concentration of common metabolic indicators of urolithiasis in urine (i.e., oxalate, citrate, uric acid, calcium, and magnesium) was developed to improve the efficiency. To validate its precision and specificity, standard curves were prepared using working solution of these indicators. 100 standard solutions of these indicators were measured with our new IC and three other ICs as the control instruments; analyte concentrations in 100 24-h urine samples from volunteers and 135 calculi patients were also measured.

**Results:**

All analytes had good linear relationships in concentration ranges of 0–10 mg/L. The precision experiments in the standard and urine samples showed that the measurement errors of the newly developed IC were all less than 5%. In urine, the recovery rate ranged from 99.6 to 100.4%, the coefficient of variation ranged from 1.39 to 2.99%, and the results matched between our newly developed IC and the control ICs. The results of the efficiency test showed that we can finish the analysis at the average number of 14 people per day with the new IC. While the average number in the control group is 3.85/day (p = 0.000).

**Conclusions:**

Overall, this multi-channel system significantly improves the efficiency of metabolic evaluation while retaining accuracy and precision.

**Supplementary Information:**

The online version contains supplementary material available at 10.1186/s12894-021-00914-4.

## Background

Urolithiasis is a multi-etiological disease resulting from a combination of environmental and genetic factors. One of the most challenging aspects of this disease is its high recurrence rate [1–3]. The recurrence rate of urolithiasis for the formation of calcium oxalate stones, for example, may be up to ≥ 13% for over 5 years. This high recurrence rate can increase patients’ agony and economic burden, and overwhelm the currently limited medical resources.^4^

The chemical origins of urolithiasis stem from an oversaturation of stone formation factors such as oxalate ions, calcium ions, and uric acid in urine, and a deficiency of stone inhibition factors such as citrate and magnesium ions [5, 6].

For most patients, an in-depth metabolic evaluation may reveal the presence of urinary stones. Metabolic evaluation of patients with urinary stones was first proposed by Pak [7, 8]. An in-depth metabolic evaluation includes the analysis of the output of stone formation factors (oxalate, calcium, and uric acid) and stone inhibition factors (magnesium and citrate) in urine collected over the course of 24 h [9, 10]. The same stone components may result from different metabolic abnormalities, and as a consequence, their prevention regimens may differ accordingly. Thus, it is necessary to clearly define specific pathogenesis through a comprehensive metabolic evaluation in each case [11]. Some reports show that prevention aimed at etiology may protect ~ 80% of patients from recurrence, and thus, with respect to patient outcome, there is a real need for increasing accessibility of in-depth metabolic evaluation [12].

In order to complete a metabolic evaluation, small organic molecules in urine, including oxalate and citrate, are measured by ion chromatography (IC), whereas other indicators may be measured by chemical methods or colorimetry. The fact that different urinary stone-related compounds (USRCs) are measured by different methods renders the metabolic evaluation of urolithiasis quite tedious and complex. Moreover, conventional single-channel IC provides low measurement efficiency and is difficult to implement in a way that meets clinical requirements [13].

Although the five major stone formation and inhibition factors indicated above have been detectable by IC for many years, the measurement efficiency of these compounds has been low [14]. In recent years, IC has become increasingly automated, which has substantially improved the efficiency of measurements. Based on this progress, our research group has designed a novel automated IC for the detection of stone formation and inhibition factors. By exploiting a three-channel design, our chromatograph covers five of the main indicators that must be measured for the metabolic evaluation of urolithiasis. Assisted by a 120-vial micro-auto-sampler, the IC completes the sample supply to the three channels within 1 min, thus ensuring simultaneous measurement of samples, reducing the time required for manual sample injection and sample rotation, and increasing work efficiency. After completion of a measurement, the central computer reads the measurement data automatically and calculates the 24-h output of the five main indicators in the urine sample (Additional file 1: Fig. 1).

Theoretically, our design facilitates the separation between the operator and the instrument. A clinical operator can complete the preparation of urine samples in 8 h, start the instrument, and print the report the next day. In this manner, our instrument can streamline metabolic evaluation and maximize its efficiency under the existing conditions, thereby laying the foundation for the popularization and widespread application of metabolic evaluation of outpatients. Presently, our instrument has obtained three patents for invention and nine utility model patents, which were nationally authorized by the P. R. China.

In order to verify the measurement precision of our novel IC instrument, we employed 3 single-channel CIC100-type IC (CIC100) manufactured by Qingdao Shenghan Chromatographic Technology Co., Ltd. as the control instrument. The CIC100 instruments passed the measurement precision test, complying with the relevant requirements of the *JJG 823–2014, Chinese Procedure for Quality Control of Ion Chromatography Instruments*.

## Methods

### Construction of an automated three-channel IC instrument

The construction of our novel IC is shown in Additional file 1: Fig. 1. The IC device consists of three independent chromatographic systems. The first IC measurement channel is equipped with an anion-exchange column (SHODEX 52-4E 4.6 × 250 mm) for the analysis of negative ions (*i.e.*, oxalic and **citrate**). The second system incorporates a cation-exchange column (SH-CC-3 4.6 × 150 mm) for the analysis of positive ions (*i.e.*, magnesium and calcium ions). The final IC analysis system is comprised of an HPLC unit with a C18 column (C18-WP 100A 4.6 × 150 mm, 5 μm) for the detection of uric acid via ultraviolet liquid chromatography. The auto-sampler completes the sample supply to the three channels within 1 min, thus ensuring simultaneous measurement of the components in all three channels. The concentrations of all five USRCs in urine samples obtained over 24 h are calculated using the Etiological-Diagnosis Software developed by our research group, by comparing the relevant sample signals with the signals arising from standards of known concentration.

To verify the measurement precision and efficiency of the new IC, 3 CIC100 were used as the control instrument. Three different chromatographic columns and their signal detectors were installed in CIC100 to measure the five target USRCs. In 3 CIC100 ICs, we replaced the all tech cation column with the SH-CC-3 cation column and the TSK-GEL Super IC-Anion column with the Shodex 52-4E column.

### Urine sample collection and processing prior to USRC determination

24-h urine samples were collected from human subjects in accordance with ethical guidelines. One hundred volunteers (74 males and 26 females; average age of 41 years, ranging from 18 to 67) without any history of urinary system diseases such as infections, hematuria, or stones, provided their 24-h urine. Prior to the measurements, the volunteers accepted a routine urine test to screen for hematuria or infections. If a patient’s sample revealed hematuria or infection, their urine was excluded from the assay.

We also selected 135 patients with stones (99 males and 36 females; average age 41.469 years, ranging from 18 to 69) and analyzed their 24-h urine samples. Prior to the measurements, the patients accepted a routine urine test to screen for hematuria or infections. If a patient’s sample revealed hematuria or infection, their urine was excluded from the assay.

25 mL of 36.5% hydrochloric acid was added to each sample when the volunteer/patient gave his/her urine for the first time after the timing point of 24-h urine collection. 100 mL of acidified urine was transferred to a 50 mL test tube for analysis of oxalic acid, citrate, magnesium and calcium ions when urine collection was completed. Subsequently, a solution of aqueous sodium hydroxide (1 g/L) was added to alkalize the urine in order to measure the uric acid levels. The pH value was adjusted from 8 to 10 and the sample of alkalized urine was subsequently allowed to stand for 30 min prior to analysis.

### IC operation

A 4.6 mM sodium carbonate eluent (flow rate of 0.8 mL/min) was prepared for the analysis of oxalic acid and citrate using the anion-exchange column (SHODEX 52-4E). Further, a 5.5 mM loprazolam eluent (flow rate of 1.5 mL/min) was prepared for the analysis of magnesium and calcium ions on the cation-exchange column (SH-CC-3). For the analysis of uric acid on the C18-WP column, an eluent consisting of 0.1% acetic acid (flow rate of 1 mL/min) was prepared. Reference standards and all other samples were prepared in containers for the automatic sampler, which were cleaned thoroughly with deionized water. Once prepared, the automatic sampler feeds the samples sequentially to the relevant analysis system. The chromatographic software then calculates the concentrations of the various components.

### Preparation of standard curves

Pure solids of sodium oxalate, sodium citrate, calcium chloride, magnesium chloride, and uric acid were analytically dissolved in deionized water to furnish five 1 g/L working solutions. Additionally, the uric acid working solution was alkalized with a sodium hydroxide solution to a pH of 8–10 to facilitate dissolution. Each working solution was diluted with deionized water to reach the target analyte concentration. Oxalate ion solutions at concentrations of 1.0, 10.0, 20.0, and 100.0 mg/L, citrate ion solutions at concentrations of 10.0, 100.0, 500.0, and 1,000.0 mg/L were prepared. Calcium ion solutions at concentrations of 10.0, 100.0, 200.0, and 500.0 mg/L, magnesium ion solutions at concentrations of 1.0, 50.0, 100.0, and 200.0 mg/L were prepared from their respective stock solutions. Lastly, urate solutions were prepared at concentrations of 10.0, 200.0, 500.0, and 1,000 mg/L. An adequate volume (2–5 µL) of each solution was transferred and injected into our new IC as well as the control ICs. Data were processed using the Chromatography Workstation Software and the standard curves were plotted automatically.

### Precision experiments performed on standards and urine samples

Each of the previously prepared working solutions was diluted with deionized water to prepare standard solutions (*i.e.*, oxalate at 20 mg/mL, citrate at 50 mg/L, magnesium ion at 30 mg/mL, calcium ion at 50 mg/mL, and urate at 100 mg/mL). Each of these standard solutions was diluted twenty times and twelve replicate measurements of each compound were performed in one day. A total of 100 standard solution samples were analyzed by the control instrument and our new instrument.

Every 24-h urine sample (including acidified and alkalized urine samples) obtained from the volunteers was diluted ten times and sent to the chromatographs belonging to the newly developed and control ICs. Urine samples of 135 patients with stones were injected into the chromatographs of the two groups to detect the concentrations of the five target USRCs, and *t*-test was used to perform statistical analysis.

### Recovery experiments

After the concentration of each of the five components was detected in every sample, recovery tests were performed. For each diluted acidified urine sample, 40 mL were divided into four equal parts. To every part, a quantity of oxalate working solution (0.05, 0.1, 0.15, 0.2, 0.25, or 0.3 mL of 100 mg/L solution), citrate working solution (0.1, 0.15, 0.2, 0.25, 0.3, or 0.35 mL of 1,000 mg/L solution), magnesium ion working solution (0.1, 0.2, 0.3, 0.4, 0.5, or 0.6 mL of 100 mg/L solution), and calcium ion working solution (0.1, 0.2, 0.3, 0.4, 0.5, or 0.6 mL of 200 mg/L solution) was added. To 10 mL of each diluted alkalized urine sample, 0.1, 0.15, 0.2, 0.25, 0.3, or 0.35 mL of 1,000 mg/L of uric acid working solution was added. The concentration of each of the components was measured by our new IC, the measured amount was determined (viz. the concentration difference of each component in the liquid samples multiplied by the volume), the recovery rate and the variation coefficient was calculated. All 100 urine samples of the volunteers were subjected to recovery analysis.

### Efficiency test

We made a statistics of the number of patients who finished the examination with each IC everyday as an efficiency test. As it is hard to evaluate the maximum operating efficiency with ordinary patients because of the random number of patient reception. We concentrated the volunteers to make the operating efficiency come close to the superior limit efficiency.

### Statistical analysis

A statistical analysis for the experimental data was carried out using the SPSS 19.0 software by performing a *t*-test analysis, using P < 0.05 as a statistically significant difference.

## Results

### Preparation of the standard curves

The peaks of oxalate, citrate, magnesium, calcium, and uric acid appeared at 13.830 min, 23.718 min, 4.411 min, 6.031 min, and 22.26 min, respectively. The data were processed by Chromatography Workstation software and the standard curves were plotted automatically. The standard curves showed good linearity in the range from 1 to 100 mg/L for oxalate with r = 0.999, an offset value (*i.e.*, *y*-intercept) of –0.105 and slope of 5.887E + 04. The detection limit was determined to be 4.10 μg/L. Meanwhile, an adequate volume of the 1 g/L working solution was diluted to 0.5, 1.0, 2.5, and 10.0 mg/L. The results displayed good linearity in the range from 0 to 10 mg/L (Additional file 2: Fig. 2). As shown in Additional file 2: Fig. 2, the results obtained for the remaining four components were similar.

### Precision experiments

The measurement results of the standard solutions are shown in Additional file 3: Fig. 3 and Table 1. The results show that the concentrations of the standard solutions, measured by both the control CIC100 and the new IC, were very similar (P > 0.05) and the measurement error attained with the new IC, as defined by the coefficient of variation, was below 5%. The sensitivity and specificity of the two sets of equipment testing standard samples were 100% since both of them could effectively detect the target components from the standard sample.

**Table 1:** Results from the precision experiments conducted with standard solutions

	3-Channel IC	Control group	*t*	P
	Concentration (mg/L)	Concentration (mg/L)		
Oxalate	0.998 ± 0.0084	0.998 ± 0.0081	− 0.024	0.981
Citrate	2.498 ± 0.0104	2.501 ± 0.0098	− 1.743	0.084
Calcium	2.498 ± 0.0075	2.500 ± 0.0099	− 1.145	0.255
Magnesium	1.500 ± 0.0060	1.499 ± 0.0086	1.400	0.165
Uric acid	4.992 ± 0.0415	4.960 ± 0.301	1.045	0.299

The results obtained from the analysis of 24-h urine samples are shown in Additional file 4: Fig. 4 and Table 2. The measurement results were consistent between the two groups (P > 0.05). Among the result of the 100 samples, only 6 of the 100 volunteers was diagnosed with hyperoxaluria. And there is no other metabolic disorder exist (Additional file 5: Fig. 5).

**Table 2:** Mean concentrations of stone-related components in all 24-h urine samples from volunteers (self-control and *t*-test)

	3-Channel IC	Control group	*T*	P
	Concentration (mg/L)	Concentration (mg/L)		
Oxalate	4.29 ± 2.21	4.32 ± 2.18	− 0.962	0.339
Citrate	408.46 ± 18.92	408.48 ± 18.87	− 0.054	0.957
Calcium	53.21 ± 2.06	53.20 ± 2.06	1.155	0.251
Magnesium	48.28 ± 2.03	48.30 ± 2.03	− 0.653	0.515
Uric acid	48.23 ± 2.42	47.62 ± 2.40	1.444	0.152

Additionally, the results of the analysis of the 24-h urine test samples are shown in Additional file 5: Fig. 5 and Table 3. The *t*-test shows that the results obtained from the two IC groups are similar, with P > 0.05. Most patients with urolithiasis had a metabolic disorder. Of 135 patients, 126 patients were found to have a variety of metabolic abnormalities (including 35 cases of low urine). Two patients did not present any metabolic abnormalities, though they also had low urine output (< 2000 mL) for over 24 h. 69 patients had high oxalic acid levels, which was the highest incidence rate. Two patients showed severely high oxalic acid levels (> 100 mg of oxalic acid excretion for over 24 h). Additionally, there were 55 cases of high urinary calcium levels, 57 cases of low urinary citrate levels, 6 case of severely low urinary citrate level (< 100 mg of citrate excretion for over 24 h), 12 cases of high uric acid levels, and 39 cases of low urinary magnesium levels. 58 patients showed more than two metabolic abnormalities and 18 patients had more than three metabolic abnormalities. The patients in this study had a wide selection of patients, covering multiple ages, with different stone positions (renal, ureter or bladder) and sizes. By examining the patient’s urine and medical history, those with infections, medical stones, history of drug use, and hereditary urinary stones were excluded from the group.

**Table 3:** Results obtained from patients with stones

	New ion chromatograph	Control Group	*t* value	P value
	Concentration (mg/L)	Concentration (mg/L)		
Oxalate	25.81 ± 19.76	25.88 ± 19.73	− 1.036	0.302
Citrate	220.63 ± 164.17	220.82 ± 164.62	− 0.268	0.789
Calcium	129.28 ± 131.49	122.34 ± 106.58	1.004	0.317
Magnesium	52.86 ± 67.89	52.87 ± 68.06	− 0.029	0.977
Uric acid	254.68 ± 220.66	253.38 ± 220.156	1.589	0.114

### Recovery experiments

The results of the recovery experiments are shown in Table 4. The recovery rates and variation coefficient of the five target constituents in 100 urine samples were comparable (P > 0.05). In view of the recovery experiment, the new IC can effectively detect the working fluid added to the urine sample. The sensitivity and specificity of the new IC for the detection of calculus-related components in urine are 100%.

**Table 4:** Mean recovery rates and coefficients of variation obtained from recovery experiments

	New ion chromatograph		Control group		Mean recovery rate		Variation coefficient (%)	
	Mean recovery rate (%)	Variation coefficient (%)	Mean recovery rate (%)	Variation coefficient (%)	*t* value	P value	*t* value	P value
Oxalate	100.5 ± 1.04	2.86 ± 0.21	100.6 ± 1.04	2.84 ± 0.19	− 1.044	0.299	0.821	0.414
Citrate	100.4 ± 1.05	1.05 ± 0.11	100.6 ± 1.17	1.04 ± 0.10	− 1.197	0.234	0.498	0.620
Calcium	100.5 ± 1.08	1.31 ± 0.29	100.4 ± 1.01	1.31 ± 0.29	0.324	0.747	0.159	0.874
Magnesium	100.6 ± 1.11	2.00 ± 0.41	100.5 ± 1.12	2.00 ± 0.41	0.478	0.634	− 0.344	0.732
Uric acid	100.5 ± 1.01	1.35 ± 0.37	100.5 ± 1.11	1.35 ± 0.38	− 0.203	0.839	− 0.366	0.715

### Efficiency test

The results of the efficiency test are shown in Additional file 6: Fig. 6. We can finish the analysis at the average number of 14 people per day with the new IC. While the average number in the control group is 3.85/day(p = 0.000).

## Discussion

The formation of urinary stones is a common and frequently occurring urological disease. Calcium oxalate stones are the most common type of urinary stones [1–4]. Urinary stones are a complex, multi-etiological disease, whose main pathogenic factors include hyperoxaluria, hypercalciuria, hypocitraturia, hypomagnesuria, and hyperuricosuria [15–17]. Thus, the core component of an in-depth metabolic evaluation is the detection of the output of USRCs in a urine sample obtained over the course of 24 h in order to identify the above-mentioned pathogenic factors. The methods employed for the analysis of USRCs during a conventional metabolic evaluation are varied and include techniques such as IC, colorimetry, and chemical methods [18]. IC is suitable for the separation of hydrophilic (negative and positive) ions. Studies have demonstrated that the precision of IC for the measurement of oxalate concentration is higher than those of other methods, and currently represents the gold standard [19, 20]. However, single-channel ICs are quite inefficient and cannot be used to measure multiple USRCs or large numbers of samples.

The measurement range available by IC has covered the above-mentioned five components for many years [14]. Recently, however, technological advances in multi-channel designs and auto-sampling techniques have gradually facilitated automation and multi-functionalization of ICs. Accordingly, our research group has developed a novel multi-channel IC instrument specifically designed for metabolic evaluation, which reduces evaluation time and allows for the automation of measurements and reports using an auto-sampler and computer software (Additional file 1: Fig. 1). However, the design and manufacture of a multi-channel IC is not a simple superposition of multiple ICs, as the presence of oscillations and electromagnetic interference may theoretically decrease measurement precision. Thus, the measurement precision of the new IC needed to be verified. Three CIC100 instruments were selected as the control, which comply with the Chinese National Testing Standard for Precision Instruments. Standard curves were prepared with the substances and precision experiments were carried out. It was obvious that the five working solutions exhibited good linear relationships (Additional file 2: Fig. 2). The results of the precision experiments showed that the measurement errors obtained for the standard solutions of all five components were within 5% with the new IC. Moreover, the measurement results obtained using the chromatographs associated with the two different groups were comparable (P > 0.05, Table 1).

The composition of urine is quite complex and the presence of multiple impurities in urine may interfere with IC measurements. In this work, we used 24-h urine samples from the volunteers. The results showed that the measurements obtained with the chromatographs belonging to the two different groups (new and control) were essentially identical. The measurement error determined for the new IC was < 5% (P > 0.05, Additional file 4: Fig. 4 and Table 2). The results obtained for the 24-h urine samples from patients with stones were also comparable (Table 3); that is, there were no significant differences (P > 0.05) between the results obtained using the CIC100 and our new IC.

In order to further verify the precision of the novel IC, we carried out recovery experiments. After the concentrations of all five components were measured, working solutions of each component were added to the urine samples. The concentration of each component was measured and converted to a measured amount, that is, the concentration difference of each component in the liquid samples multiplied by the volume, which was then used to calculate the recovery rate. The results from the recovery experiments showed that the recovery rates from the measurements by the novel IC were almost up to 100% (as shown in Table 4).

The results described above demonstrate that the novel IC has a good measurement precision and its operation is clearly less complex and more efficient than those of the control group ICs.

Additionally, we found that most patients who suffered from stones also suffered from a metabolic disorder (Additional file 5: Fig. 5 and Table 3). But the excretion of the stone related composition of people without stone is always shown in the normal range (Additional file 5: Fig. 5). These results demonstrate the potential utility of our instrument, showing that most patients require metabolic evaluation, in particular using the 24-h urine collection and analysis.

For conventional ICs, samples with large volume are needed. Prior to injection, the sample must be diluted and filtered to protect the chromatographic column. In principle, this step may increase the measurement error. As the novel IC adopts a micro-injection design, the injection volume required by the auto-sampler is only 2 μL. As a result, the urine impurities introduced into the chromatographic column may be ignored. Moreover, a built-in guard column was installed in front of the chromatographic column, thus allowing the original urine sample to be injected directly into the chromatographic column. Such direct injection reduces the measurement error and increases the measurement efficiency. As a result (Additional file 6: Fig. 6), the analytical efficiency of new IC (average of 14 patients/day) is 3–4 times that of the control group (average of 4 patients/day). This is just a primary data. Actually, its superior limit of efficiency would be much higher under full utilization.

## Conclusions

The results described herein show that the measurement precision of our new three-channel IC is comparable to those obtained with conventional ICs. The new IC, however, displays significantly higher measurement efficiency. The promotion and application of this instrument would be expected to provide a solid clinical and practical framework for the promotion and application of etiological diagnosis of urolithiasis, especially in outpatients.

The limitation of this study is that the new ion chromatograph was not used to compare the relationship between different components of patients' stones and urine abnormalities, which limited the value of this study to some extent. This is because most of the patients in this study did not receive surgical operation or treatment, and the stone specimens could not be obtained, thus the stone composition analysis could not be carried out. In further studies, multicenter study, study on the correlation between stone composition and urine metabolism abnormality, study on the efficiency and cost ratio will be carried out, to further verify the performance of this new ion chromatograph.

## Supplementary Information

**Additional file 1:** S1_Fig. Figure 1: Structure and photograph of the novel ion chromatograph.

**Additional file 2:** S2_Fig. Figure 2: Preparation of standard curves for oxalic acid, magnesium, uric acid, calcium, and citric acid(the images depicted in Fig. 2 is our own).

**Additional file 3:** S3_Fig. Figure 3: Chromatograms obtained from the standard solutions of oxalic acid, magnesium, uric acid, calcium, and citrate.

**Additional file 4:** S4_Fig. Figure 4: Chromatograms of the five components present in urine: oxalic acid, magnesium, uric acid, calcium, and citrate.

**Additional file 5:** S5_Fig. Figure 5: Metabolic disorders of patients with stones and volunteers.

**Additional file 6:** S6_Fig. Figure 6: Number of volunteers analyzed by both installations

## Data Availability

The information about where the data supporting your findings can be found in our manuscript.

## Abbreviations

USRCs: Urinary stone-related compounds
IC: Ion chromatograph
