# Supplementary figures and images for: Three-channel ion chromatograph for improved metabolic evaluation of urolithiasis
Source: BMC Urol. 2021 Nov 6;21:151. doi: 10.1186/s12894-021-00914-4 (PMC8572504; doi:10.1186/s12894-021-00914-4)

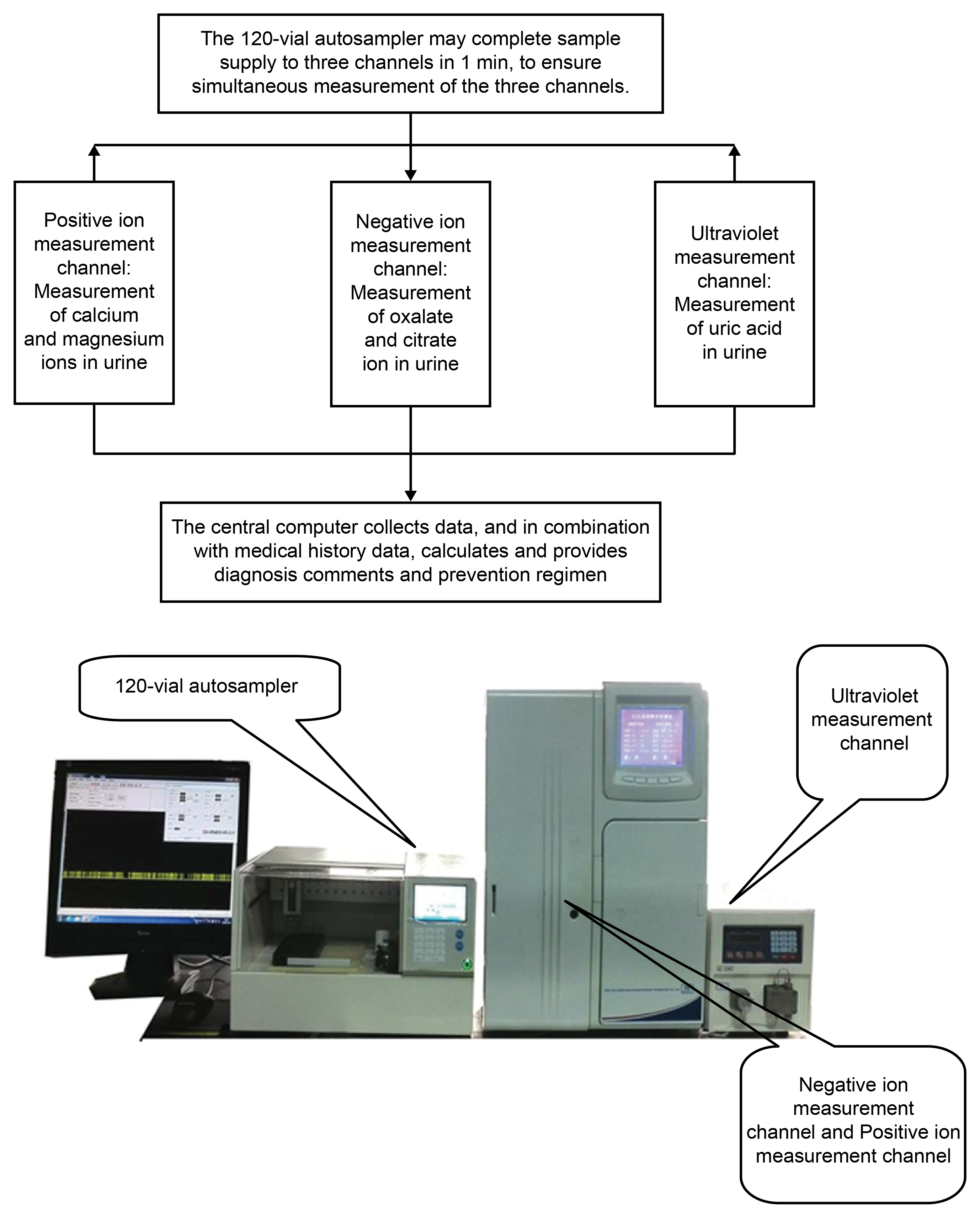

Supplement: Supplementary file 1 — Additional file 1: S1_Fig. Figure 1: Structure and photograph of the novel ion chromatograph. [file 12894_2021_914_MOESM1_ESM.tif]

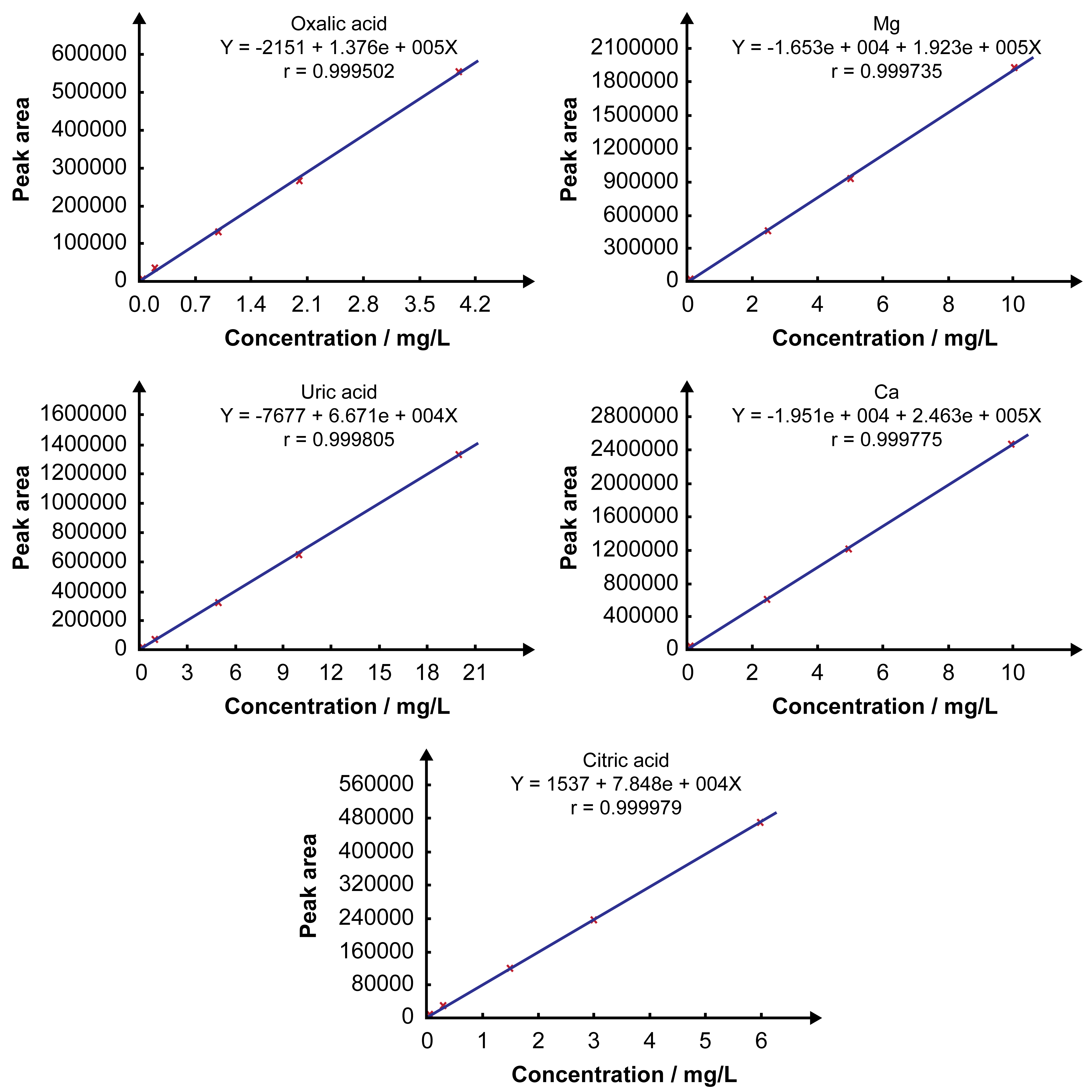

Supplement: Supplementary file 2 — Additional file 2: S2_Fig. Figure 2: Preparation of standard curves for oxalic acid, magnesium, uric acid, calcium, and citric acid(the images depicted in Fig. 2 is our own). [file 12894_2021_914_MOESM2_ESM.tif]

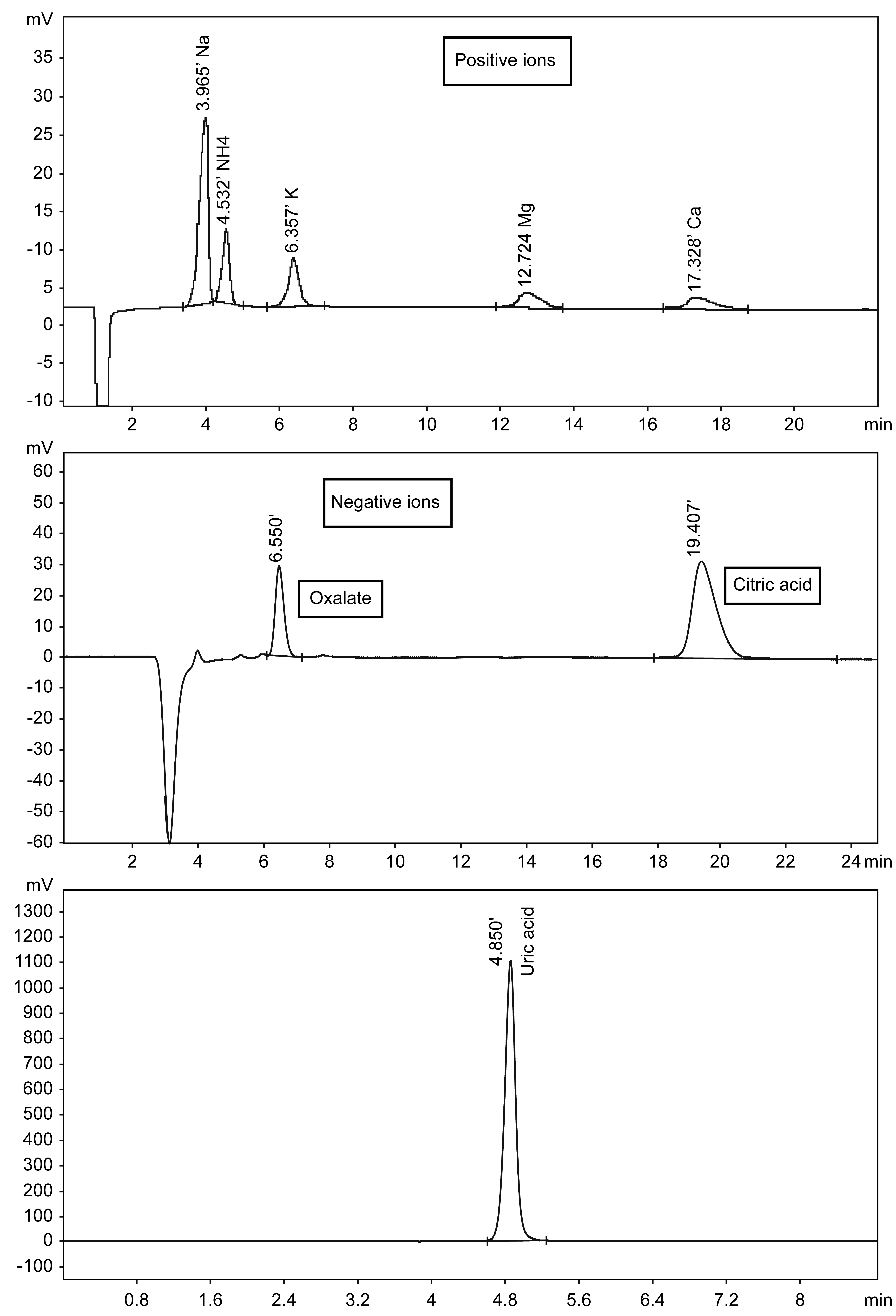

Supplement: Supplementary file 3 — Additional file 3: S3_Fig. Figure 3: Chromatograms obtained from the standard solutions of oxalic acid, magnesium, uric acid, calcium, and citrate. [file 12894_2021_914_MOESM3_ESM.tif]

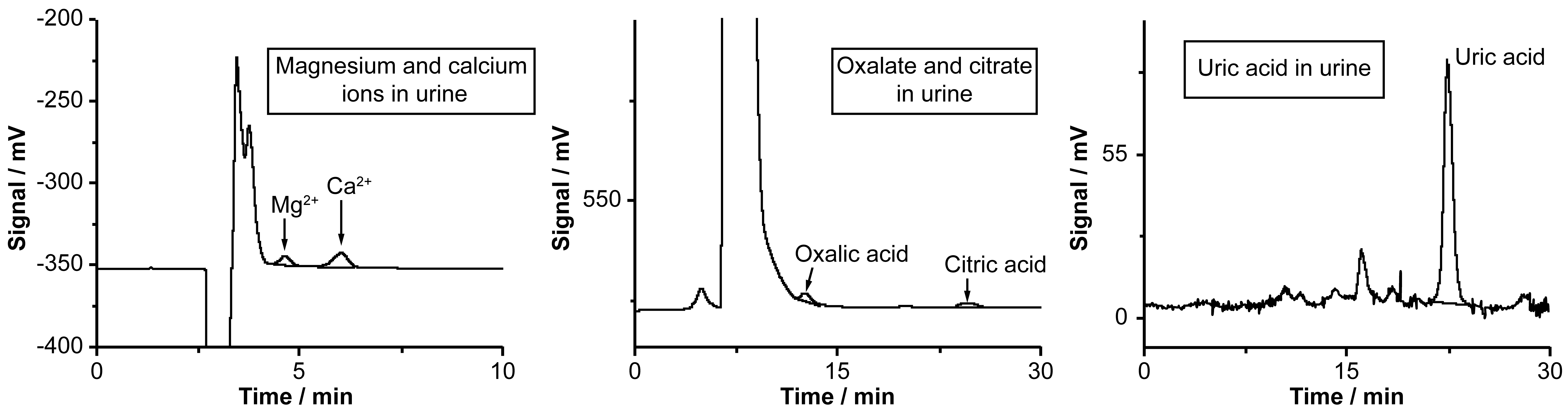

Supplement: Supplementary file 4 — Additional file 4: S4_Fig. Figure 4: Chromatograms of the five components present in urine: oxalic acid, magnesium, uric acid, calcium, and citrate. [file 12894_2021_914_MOESM4_ESM.tif]

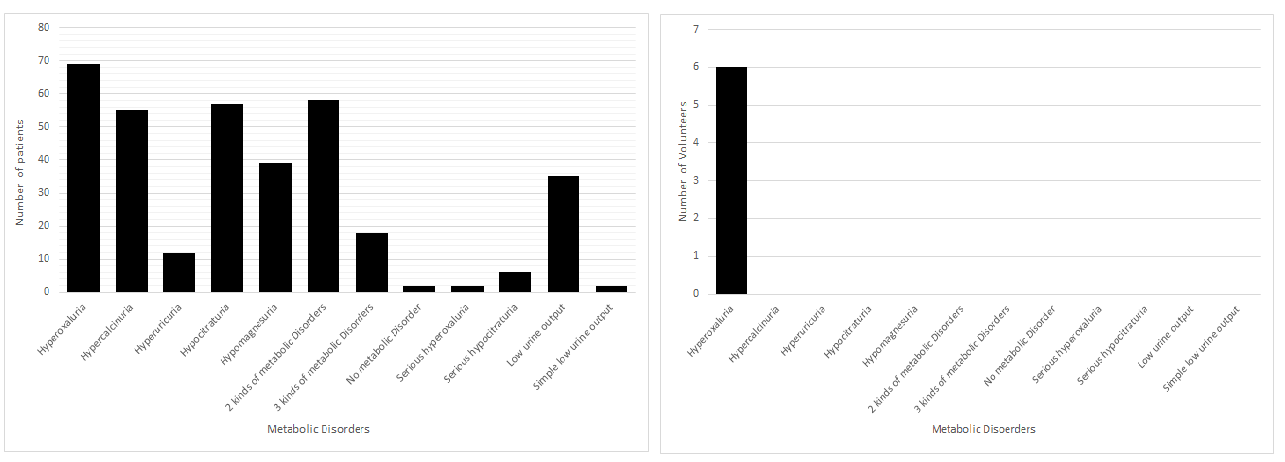

Supplement: Supplementary file 5 — Additional file 5: S5_Fig. Figure 5: Metabolic disorders of patients with stones and volunteers. [file 12894_2021_914_MOESM5_ESM.tif]

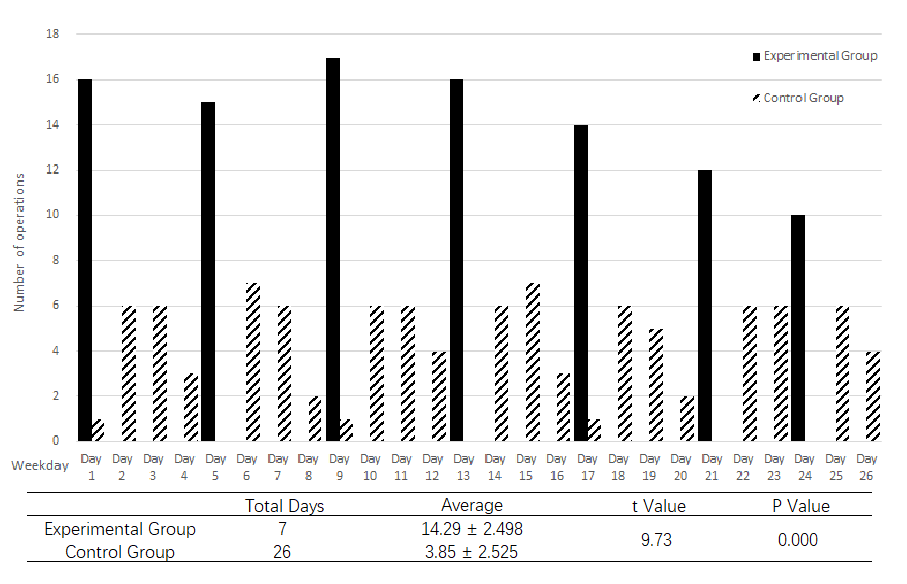

Supplement: Supplementary file 6 — Additional file 6: S6_Fig. Figure 6: Number of volunteers analyzed by both installations [file 12894_2021_914_MOESM6_ESM.tif]
